# Sense of coherence in students while studying abroad

**DOI:** 10.4102/hsag.v29i0.2585

**Published:** 2024-09-30

**Authors:** Claude-Hélène Mayer, Jeff Larsen

**Affiliations:** 1 Department of Industrial Psychology and People Management, School of Management, University of Johannesburg, Johannesburg, South Africa; 2 University for Digital Technologies in Medicine and Dentistry, Wiltz, Luxembourg; 3 Hewlett Packard Enterprise, Fort Collins, Colorado, United States

**Keywords:** sense of coherence, mental health, study abroad, semester at sea, salutogenesis

## Abstract

**Background:**

In the 1970s, medical sociologist Aaron Antonovsky developed the concept of salutogenesis, which is the study of health and development focusing on sense of coherence (SOC). Although salutogenesis has been well researched in higher education contexts, the concept has not been studied in depth in intercultural and cross-cultural settings, such as the one provided by semester at sea (SAS), which is a United States (US) study-abroad programme.

**Aim:**

The aim was to investigate and compare levels of SOC in students in the SAS study-abroad programme.

**Setting:**

The research was conducted during a selected voyage at sea over a period of 106 days.

**Methods:**

The authors used a quantitative cross-sectional correlational design and investigated mean score differences in three SOC sub-scale scores measuring meaningfulness, comprehensibility, and manageability. The authors compared US students’ SOC scores to those of students from other countries and compared the scores of women to men. Measurement invariance was firstly established before investigating mean score differences. Data were collected in the form of a survey, using Antonovsky’s 29-item Life Orientation Questionnaire.

**Results:**

The study shows that overall scores in meaningfulness were the highest, followed by manageability and comprehensibility, which potentially supports the idea that the motivational component in life of these students is the most important. Finally, women scored higher in meaningfulness than men.

**Conclusion:**

The study can provide insights in SOC in students and might provide implications for interventions regarding SOC results across diverse student populations in SAS.

**Contribution:**

This article contributes to SOC research in study-abroad programmes.

## Introduction

Semester at sea (SAS) is a United States (US) study-abroad programme that aims to develop a global perspective in students during their time abroad (Kang 2018; McCabe 1994). The SAS ship has cruised worldwide since 1963, introducing students to different cultures while they earn degree credits (Kang 2018) and develop a deeper cultural understanding and intercultural competencies (Mayer, Surtee & Visser 2016).

Longitudinal research shows that the SAS programme of studying abroad has long-term benefits for the participants, stimulating a global mindfulness and cultural sensitivity (Medora, Roy & Brown 2020). Dukes et al. (1994) found that course participants developed a strong sense of purpose in life. Another study showed that 10 years after completing the voyage, participants still ascribed meaning to the voyage and defined it as an important experience during their lifetime development (Dukes, Johnson & Newton 1991). Dukes (2006:209) found that 22 years after the experience of the SAS, students saw the voyage as a meaningful experience and a ‘springboard for personal growth’. So far, researchers have not yet investigated the sense of coherence (SOC) and mental health of SAS students.

Researchers have investigated salutogenesis, the study of (mental) health development, for the last four decades (e.g. Antonovsky 1979; Mayer 2011; Pretorius, Walker & Heyns 2009). Sense of coherence, as the major component of salutogenesis, has been researched in various contexts including politics, education, business, and health (Maass et al. 2022; eds. Mittelmark et al. 2021), in diverse societies such as Scandinavia (Eriksson, Sagy & Lindström 2012; Saloniemi et al. 2014), Israel (Braun-Lewensohn, Abu-Kaf & Kalagy 2017), and South Africa (Louw & Mayer 2014; Matumba 2020; Strümpfer 1995). Aaron Antonovsky, a medical sociologist (1993), emphasises that individuals’ health is strongly influenced by their inner attitudes towards the world and their life, in addition to external factors such as the political system, the environment, war, or starvation. Salutogenesis predicts that individuals with many resources and strong coping strategies usually manage stressors better than others (Mayer 2011). The most important component of mental health is therefore defined as SOC, a life-orientation, which contributes to keeping individuals mentally healthy (Mittelmark 2022).

The three major aspects of SOC are: (1) comprehensibility, (2) manageability, and (3) meaningfulness. These provide measures of how people understand the world, manage resources, and create meaning in and motivate their lives (Antonovsky 1979; Mayer, Louw & Von Der Ohe 2019). Researchers have debated how SOC develops and up to what age a person can develop it. Antonovsky (1979) believed that it is best developed before the age of 30. Others, however, have found that SOC can be developed throughout life (Bahrs & Matthiessen 2007; Mayer & Krause 2011; Meier Magestretti 2022), and that it is dynamic and can change during the lifespan in connection with specific incidents (Kähönen et al. 2012). The question concerning its development is relevant to how SOC develops in university students and in higher education institutions (HEIs).

### Sense of coherence in students

Antonovsky (1979, 1987) describes SOC, which entrenches a person’s individual, socio-cultural and historical contextual experience, as a major health indicator that buffers stressors in daily life. Researchers have found a strong relationships between SOC and the development of intercultural competencies, meaning-making, overcoming challenges, finding solutions to and managing conflicts in complex situations, physical health, and resilience (Mayer 2011; Mayer & Boness 2009, 2013; Mayer & Krause 2011; Morrison & Clift 2006). Therefore, SOC influences a person’s health and well-being (Daoud et al. 2014; Lindström & Eriksson 2010).

Research has shown that students in HEIs increasingly experience challenges with mental health, such as increased stress, anxiety, depression, and burnout (Gallego et al. 2014; Regehr, Glancy & Pitts 2013), especially since the coronavirus disease 2019 (COVID-19) pandemic (Holm-Habdulla et al. 2022), with concomitant general health problems (Lin & Huang 2012). Higher education institutions themselves are required to be innovative, internationalised, and responsive to change (Fan et al. 2017). They must also monitor and promote staff and students’ health and well-being, since burnout and mental ill-health in staff and students are reportedly on the rise (Dooris, Doherty & Orme 2017; Mayer & Boness 2011). Furthermore, international researchers confirm that students’ mental health is worsening (Campbell et al. 2022). Strategies to improve mental health and well-being focus on increasing supportive social networks, preparing students for their studies, and training them to adapt to new social and cultural contexts (Campbell et al. 2022).

Salutogenesis research in teaching and learning – as summarised in De Oliveira Olney and Kiss’s (2022) well-researched overview – has shown that a strong SOC in students can support their ability to cope with complex experiences and simultaneously stay healthy (Bracha & Bocos 2015; Brewer et al. 2019; Darling et al. 2007; Dooris, Wills & Newton 2014; Garista, Pocetta & Lindström 2019; Leela, Loo & Tan 2021; Louw et al. 2019; Matić et al. 2022; Pretorius et al. 2009; Suraj & Singh 2011). Higher education institutions are often complex in terms of culture and socio-cultural student backgrounds, and as international institutions, they expose students to a diversity of experiences (Killick 2017). A strong SOC is needed to deal with diversity, interculturalisation and internationalisation effectively and healthily (Jakovljevic 2018).

### Sense of coherence in the context of culture and interculturality

Studies have shown that culture influences SOC (Antonovsky 1979, 1987; Ying, Lee & Tsai 2007), and that SOC’s development affects intercultural competencies while being simultaneously influenced by intercultural competencies (Mayer 2011; Mayer & Oosthuizen 2020; Olney & Kiss 2022). For example, Crowther and Lau (2019) demonstrate that the SOC of Polish migrant women in Scotland helped them to understand their unique experiences, and challenges with intercultural communication, and their ability to comprehend situations in the context of their own cultural values, psychosocial experiences, and beliefs. Therefore, SOC is an important consideration that might help people to cope in a cultural context and deal with their vulnerability (Crowther & Lau 2019).

Researchers have investigated SOC in university students from diverse backgrounds, such as students from different cultural or national contexts (Chu et al. 2016). In the educational system, SOC shows in better academic performance, achievement, and learning success (Mayer & Boness 2011). Researchers have also shown that SOC is associated with healthier behaviours, fewer health complaints, less worry, higher well-being, and reduced emotional loneliness in university students (Binkowska-Bury & Januszewicz 2010; Dadaczynski et al. 2022; Nosheen, Naveed Riaz & Batool 2014; Suraj & Singh 2011). These benefits suggest that universities should implement programmes to increase students’ SOC. However, the development of SOC might differ across student cultural backgrounds. For example, a comparative study of German and Chinese students showed that German students scored higher in total SOC and in its sub-categories, especially meaningfulness. In both groups, female students scored higher than male students (Louw et al. 2019). A recent study from Japan (Omiya et al. 2022) showed that educational systems should consider gender differences in SOC.

A study by He (2019) showed that SOC is related to students’ intercultural abilities, where a high SOC is negatively related to the avoidance-orientated strategies of international students coping in a new cultural academic environment. Ying et al. (2000) found that racial and ethnic similarity might be associated with SOC development. These researchers reported that:

[*I*]ndividuals with a racially/ethnically mixed network enjoyed the highest sense of coherence, followed by those with an ethnically same network, and those with either a racially same or mixed network reported the lowest sense of coherence. (p. 8)

Da-Silva-Domingues et al. (2022) point out that SOC research should consider cultural diversity and educational context to understand SOC and determine the relationships between SOC and health-related behaviours. As previously suggested, HEIs might consider programmes to promote students’ SOC in order to prepare them for work, careers, unexpected changes, and to build or maintain positive health (Dadaczynski et al. 2022; Dooris et al. 2017; Krause & Mayer 2012).

## Research methods and design

### Study setting

The study was conducted during an SAS voyage in Fall 2022 and included the total of 357 SAS students boarding the selected voyage. The study used a cross-sectional correlation design. The participants were requested to participate by completing Antonovsky’s (1987) Life Orientation Questionnaire in the beginning and at the end of the SAS voyage.

### Data collection

The data were collected using Antonovsky’s (1987) Life Orientation Questionnaire, which consists of 29 items and uses a seven-point semantic differential response format. In addition, the questionnaire has three sub-scales measuring comprehension (11 items), management (10 items), and meaningfulness (9 items). The authors reverse-scored the necessary items following the scoring instructions before calculating the participants’ mean total SOC and three sub-scale scores.

### Data analysis

The authors analysed the data using *R* version 4.3.1 (R Core Team 2023). They used independent samples *t*-tests to investigate mean score differences in the SOC sub-scale scores for US versus other participants and women versus men participants. Because scale scores must be bias-free before comparing mean scores, the authors first fitted the graded response model (Samejima 1969) to the item responses and then investigated differential test functioning (DTF) to ensure that the groups’ scale scores were comparable (Chalmers, Counsell & Flora 2016). The authors used ordinal logistic regression in the *lordif* package version 0.3–3 to identify items with differential item functioning. Items with no differential item functioning as achoring items were used in the mirt package version 1.3.9 to investigate DTF (Chalmers 2012). The authors treated the latent means and variances as free estimates in the focal group. The DTF approach provides a signed and unsigned test value. The former indicates systematic test bias, and the latter indicates average bias. It also provides a standardised effect size that indicates the percentage score difference between groups across a test. This effect size will be 0 when no DTF is present (Chalmers et al. 2016).

### Ethical considerations

Ethical considerations as detailed by Charmaz (2011) and Englander (2016) were followed during the research. These were the need for anonymity, confidentiality, voluntary participation, informed consent, and the right of the participant to withdraw from the research study at any point. All participants were orally informed that the data will be used for research purposes and published. Ethical clearance to conduct this study was obtained from the University for Digital Technologies in Medicine & Dentistry (DTMD).

## Results

Altogether, 357 students participated in this study. The participants’ mean age was 21.29 years (median = 21, s.d. [standard deviation] = 5.28). Most participants resided in the US (*n* = 280, 83%). The remainder (*n* = 59, 17%) were from different countries, such as Belgium, Chile, Canada, and Mexico. Most participants were women (*n* = 256, 73%) or men (*n* = 95, 27%). Six participants (2%) identified as non-binary.

Table 1 presents descriptive statistics for the Life Orientation Questionnaire scores for the combined group of US versus other countries participants and women versus men.

**TABLE 1:** Descriptive statistics of the Life Orientation Questionnaire scores for nationality and gender.

Scale	Mean	s.d.	Median	Skewness	Kurtosis	s.e.
Comprehensibility	3.86	0.78	3.91	−0.18	0.16	0.04
Manageability	4.80	0.85	4.9	−0.39	0.5	0.05
Meaningfulness	5.17	0.93	5.12	−0.42	0.46	0.05
**Nationality United States (*n* = 280)**						
Comprehensibility	3.90	0.75	3.91	0.08	−0.11	0.04
Manageability	4.80	0.81	4.85	−0.06	−0.12	0.05
Meaningfulness	5.19	0.90	5.12	−0.35	0.51	0.05
**Nationality other countries (*n* = 59)**						
Comprehensibility	3.69	0.89	3.82	−0.69	−0.18	0.12
Manageability	4.88	0.99	5.1	−1.09	1.45	0.13
Meaningfulness	5.10	1.06	5.12	−0.61	0.31	0.14
**Gender women (*n* = 256)**						
Comprehensibility	3.90	0.78	3.91	−0.02	−0.24	0.05
Manageability	4.84	0.83	4.90	−0.17	−0.26	0.05
Meaningfulness	5.24	0.89	5.25	−0.22	−0.30	0.06
**Gender men (*n* = 95)**						
Comprehensibility	3.79	0.75	3.73	−0.56	0.93	0.08
Manageability	4.74	0.90	4.80	−0.86	1.8	0.09
Meaningfulness	4.93	1.01	4.88	−0.61	1.13	0.10

Note: US coded 0 and other countries coded 1. Women coded 0 and men coded 1.

s.d., standard deviation; s.e., standard error of the mean.

### Comprehensibility

Table 2 presents the comprehensibility scale GRM item parameters and fit statistics. The model had a satisfactory fit to the data (*M*^2^[44] = 118.52, *p* < 0.001, RMSEA [root mean square error of approximation] = 0.069, SRMSR = 0.07, TLI = 0.90, CFI = 0.92). The items’ factor loadings ranged from 0.28 to 0.68, and the mean slope parameter was 1.03 (median = 0.91, s.d. = 0.34). No items showed statistically significant misfit based on the signed chi-squared test. The scale score alpha and marginal reliability coefficients were 0.75 and 0.79.

**TABLE 2:** Comprehension graded response model item parameters and fit statistics.

Item	*λ*	A	b_1_	b_2_	b_3_	b_4_	b_5_	b_6_	G	*χ^2^*	*df*	RMSEA	*p*
C1	0.46	0.89	−4.86	−3.36	−1.78	−0.67	0.43	3.05	−1.28	9.80	88	0.01	0.398
C3	0.28	0.50	−9.15	−4.17	−1.93	0.78	2.96	6.16	−0.64	104.79	95	0.02	0.231
C5	0.35	0.64	−5.42	−2.72	−0.32	1.29	3.24	7.30	0.44	89.66	93	0.00	0.579
C10	0.54	1.09	−1.57	−0.57	0.33	1.34	2.59	4.37	0.94	107.74	97	0.02	0.214
C12	0.59	1.24	−3.64	−2.19	−0.94	0.05	1.25	2.83	−0.45	66.30	80	0.00	0.864
C15	0.45	0.85	−4.16	−2.80	−1.38	0.86	2.91	5.20	−0.13	73.25	79	0.00	0.661
C17	0.47	0.91	−2.37	−0.97	0.39	2.04	3.60	5.35	1.27	96.03	90	0.01	0.312
C19	0.68	1.57	−1.82	−0.80	0.05	0.94	1.88	2.94	0.51	82.11	87	0.00	0.628
C21	0.58	1.20	−2.51	−1.22	−0.16	0.73	1.48	2.70	0.21	81.88	100	0.00	0.907
C24	0.68	1.57	−2.14	−0.97	0.04	1.00	1.73	3.10	0.48	108.93	80	0.03	0.017
C26	0.46	0.88	−3.16	−1.82	−0.68	0.96	2.08	3.45	0.14	97.74	105	0.00	0.680

λ, factor loadings; a, item slope; b, item thresholds; G, generalised item difficulty; *df*, degrees of freedom; RMSEA, root mean square error of approximation.

Item C10 showed uniform differential item functioning for nationality (*p* = 0.001). Therefore, the authors used all the other items as anchors in a multigroup analysis. The signed DTF between -5 and 5 on the latent trait was statistically significant (*p* = 0.027) with an unsigned DTF effect size of 0.54% (0.26%, 1.33%). Consequently, the authors removed item C10 for the mean score difference test for nationality. The group’s mean score difference was not statistically significant (*t*[75.76] = 1.25, mean US = 3.96, mean other countries = 3.80, mean score difference = 0.16 [-0.07, 0.41], *p* = 0.216, *d* = 0.20 [-0.11, 0.53]).

The comprehension scale scores had no differential item or test functioning across gender. The group’s mean score difference was also not statistically significant (*t*[173.69] = 1.19, mean women = 3.90, mean men = 3.79, mean score difference = 0.11 [-0.07, 0.29], *p* = 0.236, *d* = 0.14 [-0.08, 0.38]).

### Manageability

Table 3 presents the manageability scale GRM item parameters and fit statistics. The model had a satisfactory fit to the data (*M*^2^[35] = 72.69, *p* < 0.001, RMSEA = 0.055, SRMSR = 0.06, TLI = 0.95, CFI = 0.96). The items’ factor loadings ranged from 0.34 to 0.78, and the mean slope parameter was 1.21 (median = 1.21, s.d. = 0.45). No items showed statistically significant misfit based on the signed chi-squared test. The scale score alpha and marginal reliability coefficients were 0.76 and 0.82.

**TABLE 3:** Manageability graded response model item parameters and fit statistics.

Item	*λ*	A	b_1_	b_2_	b_3_	b_4_	b_5_	b_6_	G	*χ^2^*	*df*	RMSEA	*p*
MA2	0.45	0.85	−4.97	−3.36	−2.02	−0.63	0.79	3.06	−1.28	77.80	88	0.00	0.773
MA6	0.40	0.75	−4.09	−2.55	−0.63	0.87	2.56	6.32	0.14	97.97	88	0.02	0.219
MA9	0.34	0.62	−5.99	−3.47	−2.02	−0.58	1.05	4.15	−1.23	103.44	102	0.01	0.441
MA13	0.65	1.46	−3.36	−2.76	−2.03	−1.18	−0.41	1.09	−1.55	85.81	70	0.03	0.096
MA18	0.48	0.94	−2.86	−1.73	−0.91	0.01	1.09	2.38	−0.37	94.78	102	0.00	0.681
MA20	0.69	1.62	−3.57	−2.27	−1.39	−0.65	0.14	1.36	−1.04	8.87	78	0.01	0.390
MA23	0.58	1.21	−3.33	−2.62	−1.95	−1.27	−0.76	0.20	−1.63	74.95	82	0.00	0.697
MA25	0.61	1.30	−2.78	−1.67	−0.65	0.16	1.37	2.80	−0.20	91.78	80	0.02	0.173
MA27	0.78	2.10	−3.00	−2.40	−1.72	−0.95	−0.02	0.96	−1.29	65.63	58	0.02	0.230
MA29	0.58	1.22	−3.43	−2.23	−1.09	−0.25	0.36	1.77	−0.77	94.68	92	0.01	0.403

λ, factor loadings; a, item slope; b, item thresholds; G, generalised item difficulty; *df*, degrees of freedom; RMSEA, root mean square error of approximation.

No items showed differential item functioning for nationality and gender. The nationality group’s mean score difference was not statistically significant (*t*[75.33] = -0.56, mean US = 4.80, mean other countries = 4.88, mean score difference = -0.08 [-0.32, 0.22], *p* = 0.578, *d* = -0.09 [-0.40, 0.26]). The gender group’s mean score difference was also not statistically significant (*t*[157] = 0.94, mean women = 4.84, mean men = 4.74, mean score difference = 0.01 [-0.10, 0.32], *p* = 0.350, *d* = 0.12 [-0.12, 0.37]).

### Meaningfulness

Table 4 presents the meaningfulness scale GRM item parameters and fit statistics. The model had a satisfactory fit to the data (*M*^2^[20] = 71.61, *p* < 0.001, RMSEA = 0.085, SRMSR = 0.07, TLI = 0.94, CFI = 0.95). The items’ factor loadings ranged from 0.35 to 0.80, and the mean slope parameter was 1.53 (median = 1.51, s.d. = 0.54). No items showed statistically significant misfit based on the signed chi-squared test.

**TABLE 4:** Meaningfulness graded response model item parameters and fit statistics.

Item	*λ*	A	b_1_	b_2_	b_3_	b_4_	b_5_	b_6_	G	*χ^2^*	*df*	RMSEA	*p*
ME4	0.35	0.64	−6.89	−4.32	−2.35	−0.71	0.71	2.87	−1.67	79.33	80	0.00	0.500
ME7	0.60	1.26	−3.36	−2.50	−1.91	−0.86	0.01	1.27	−1.30	83.76	69	0.03	0.109
ME8	0.60	1.26	−3.54	−2.49	−1.89	−0.92	0.22	1.62	−1.27	76.95	65	0.02	0.147
ME11	0.57	1.18	−4.31	−3.28	−2.68	−1.48	−0.23	1.01	−1.93	65.31	58	0.02	0.238
ME14	0.76	1.97	−2.31	−2.05	−1.56	−0.83	−0.20	0.70	−1.12	69.29	63	0.02	0.274
ME16	0.72	1.75	−3.39	−2.35	−1.55	−0.58	0.54	1.90	−1.02	36.51	51	0.00	0.937
ME22	0.80	2.28	−3.02	−2.56	−2.00	−1.06	−0.44	0.39	−1.50	45.39	45	0.01	0.456
ME28	0.75	1.91	−2.65	−1.64	−0.95	−0.21	0.33	1.32	−0.61	64.12	64	0.00	0.472

λ, factor loadings; a, item slope; b, item thresholds; G, generalised item difficulty; *df*, degrees of freedom; RMSEA, root mean square error of approximation.

Item ME11 showed statistically significant uniform (*p* = 0.001) and non-uniform (*p* < 0.001) differential item functioning for nationality. Therefore, the authors used all the other items as anchors in a multigroup analysis. The signed DTF between -5 and 5 on the latent trait was not statistically significant (*p* = 0.497) with an unsigned DTF effect size of 0.97 (0.38, 1.63). Therefore, the authors did not remove this item from the nationality mean score difference test. The nationality group’s mean score difference was not statistically significant (*t*[76.71] = 0.64, mean US = 5.19, mean other countries = 5.06, mean score difference = 0.01 [-0.18, 0.40], *p* = 0.522, *d* = 0.10 [-0.21, 0.41]).

Item ME4 showed uniform differential item functioning for gender (*p* < 0.001). Therefore, the authors used all the other items as anchors in a multigroup analysis. The signed DTF between -5 and 5 on the latent trait was not statistically significant (*p* = 0.140) with an unsigned DTF effect size of 0.54 (0.17, 1.91). Therefore, the authors did not remove this item from the gender mean score difference test. The gender group’s mean score difference was statistically significant (*t*[151.95] = 2.63, mean score women = 5.24, mean score men = 4.93, mean score difference = 0.31 [0.09, 0.55], *p* = 0.010, *d* = 0.33 [0.09, 0.55]).

## Discussion

This study contributes to determining the profile of SOC sub-scales in students attending the SAS study-abroad programme from September to December 2022 to address the gap in research on SOC and mental health of this specific cohort. The research appears to be urgently needed because mental health and well-being has become an increasingly pressing issue in HEIs and for students who have been experiencing stress, depression and a general decrease in mental health (Chen et al. 2013; Gumz et al. 2014; Holm-Habdulla et al. 2022).

The SOC can change over time and with the impact of specific life events (Kähönen et al. 2012) and it can be developed throughout life (Bahrs & Matthiessen 2007; Mayer & Krause 2011; Meier Magestretti 2022). This study shows that, overall, the SAS students scored highest in meaningfulness, the component that is mainly responsible for motivation and for seeing life as worth living. This result is limited, based on the fact that they are raw scores rather than norm scores, which is the most basic form of test score. By the end of the voyage, it can be assumed that the students would have had many positive intercultural and diverse experiences, which would have contributed to their experience of meaningfulness. Involvement in shipboard culture may have prepared them well for creating meaning, and for understanding and managing their experiences and creating resilience during their time on the ship and while visiting different countries (Mayer 2011; Mayer & Boness 2009, 2013; Mayer & Krause 2011; Morrison & Clift 2006).

Furthermore, it can be assumed that the students who were on the voyage already had high levels of manageability (e.g. having organised their study marks, application forms and funding) and comprehensibility (understanding how to study in the context of the ship culture and in the context of a diverse student body). The students also underwent health checks and mental health checks to be able to join the voyage and it is further assumed that this ensured that the students who were accepted on the ship had acceptable levels of mental and physical health. It is likely that they began the SAS semester with high levels of SOC and were therefore able to cope with the complex cultural and intercultural experiences and simultaneously stay healthy (Bracha & Bocos 2015).

The students from countries other than the US also were likely to have a high manageability score to cope with the majority culture of SAS and the US university system. It can be assumed that the entire ship’s culture was strongly influenced by the US HEI system. That said, there were no statistically significant mean score differences in the three SOC sub-scales between US and non-US participant groups.

Altogether, female students scored higher than male students in meaningfulness. This finding supports similar findings in previous studies (Louw, Mayer & Surtee 2014).

This study supports the study by Ying et al. (2000), showing that students in diverse and intercultural contexts could score high in SOC based on their diverse experiences.

### Limitations

The results need to be viewed, interpreted, and understood in the context of the study’s limitations. Firstly, the study was limited to only one set of data, which was collected towards the end of one voyage. Therefore, no comparisons could be made between data from the beginning and the end of the voyage. Such comparisons would have provided better evidence for a change in SOC as a result of the SAS experience. Secondly, the study was based on a relatively small sample, which limited the type of statistical analysis conducted. Specifically, small sample sizes lead to large standard errors of the different estimates. These larger standard errors mean that the estimates are less precise and there might not be enough statistical power to detect statistical significance. Thirdly, given the biographical information of the sample, there is a bias because most of the students were from the US and defined themselves as female. This limitation means that the results might not generalise to the wider population. However, they could generalise to future SAS student populations.

## Conclusion and recommendations

This study contributes to the body of research on SOC and students in HEIs and in study-abroad programmes, especially SAS. The study’s aim was to explore the SOC of students towards the end of the SAS September–November 2022 voyage regarding overall scores and in connection with nationality and gender. The study shows that overall scores in meaningfulness were the highest, followed by manageability and comprehensibility, which potentially supports the idea that the motivational component of SOC is the most important in the lives of these students. Finally, women scored higher in meaningfulness than men.

Future research on SAS programmes should focus on comparing students’ SOC scores from the beginning of the voyage and the end of the voyage, to develop an idea of how the experience of the programme affects SOC in students who engage in such richly diverse experiences. In this context, not only data on SOC should be collected but also, for example, data on intercultural competence and other sociocultural and contextual components to develop a deeper understanding of how SOC, diversity experiences and experiences of cultural differences and similarities affect each other.

In addition, measures should be used that explore students’ experiences of being part of majority and minority cultures on SAS voyages. The fact that SAS is a US study-abroad programme could influence how students experience their being, learning, and studying on board ship and while visiting various countries. These future results could be of help in intercultural encounters in different HEIs and work contexts.

Future studies should also compare SAS students’ SOC with SOC in students from other international study-abroad programmes. This would establish whether there are differences in SOC, diversity management, intercultural competence, and majority–minority experiences between SAS and similar programmes. Furthermore, future studies need to additionally focus on the experiences of students during their time at sea and during the country visits. This could provide deeper insight into the effects of the ship’s diverse culture as opposed to experiences of another country’s culture.

On a practical note, the authors recommend that HEIs increasingly focus on the benefits of students attending study-abroad programmes such as SAS and objectively explore the impact of such programmes on students’ SOC, diversity management, and intercultural competencies. By focusing on healthy and diverse learning environments, HEIs can contribute to holistic learning, mental health and personal growth of students to prepare them for the future work and leadership environments.
